# Impact of and Comparative Outcomes for Digital and In‐Person Interventions for Complex Obesity in a Diverse Urban Population

**DOI:** 10.1002/edm2.70215

**Published:** 2026-04-03

**Authors:** Majella O'Keeffe, Emiliano Pena‐Altamira, Sumaya Shuriye, Danielle Dunk, Oliver Canfell, Rhys White, Alastair Duncan

**Affiliations:** ^1^ School of Food and Nutritional Sciences University College Cork College Road Ireland; ^2^ Department of Nutritional Sciences King's College London London UK; ^3^ Department of Nutrition and Dietetics, Guys and St Thomas' NHS Foundation Trust London UK

**Keywords:** behaviour change, ethnicity, obesity, total meal replacement, virtual, weight management

## Abstract

**Introduction:**

Obesity management in the UK includes multicomponent weight and behavioural interventions delivered at Tier 3 of the NHS model of care. This study presents the impact of and comparative outcomes for the Tier 3 Southeast London Healthy Living programme (SELHLP) in a diverse population.

**Methods:**

The SELHLP is a multicomponent, multidisciplinary programme that includes face‐to‐face (F2F) and virtual (V) delivery. Two management strategies are offered: Balance, a weight behavioural intervention and Kickstart, a three‐month total meal replacement intervention followed by a weight and behavioural intervention. Eligibility criteria were adults (≥ 18 years) with a BMI ≥ 35 kg/m^2^. Baseline data and exit data were collected at session 9–12. Primary outcomes included weight change from baseline to programme completion; secondary outcomes included both clinical and behavioural outcomes.

**Results:**

Programme completers of both Balance and Kickstart were predominantly female (82% and 79% respectively). Among completers for Balance, weight change from baseline was −2.8 (8.4) kg for Balance F2F (*p* < 0.001) and −5.1 (11.7) kg for Balance V (*p* < 0.001). A similar trend was observed for Kickstart F2F −11 (13) kg and Kickstart V −11 (11) kg (*p* < 0.001) but did not differ between service delivery models (*p* > 0.005). 34% of participants of the combined Balance and Kickstart programmes lost ≥ 5% of initial weight, −10.7% and −18% weight loss respectively. Black participants and women were less likely to achieve ≥ 5% weight loss in both programmes, but deprivation status had no effect.

**Conclusion:**

The SELHLP resulted in significant weight loss, but weight loss varied by service delivery, ethnicity and sex. Future efforts should focus on cultural salience and digitisation of Tier 3 programmes to support better engagement and completion among participants of Black ethnicity. Tailoring of Tier 3 interventions and triaging of participants at the onset should be investigated to improve both patient and service outcomes.

## Graphical abstract

The Southeast London Healthy Living Programme is a multi‐component, multi‐disciplinary Tier 3 obesity management service with virtual and face‐to‐face service delivery models. It offers two treatment streams, a behavioural weight management programme, Balance, and a total meal replacement plus a behavioural weight management programme, Kickstart. Among programme completers, significant weight loss was recorded across service delivery models and programme streams, with greater reductions observed in the Kickstart programme. Variations in programme completion and weight loss differed by ethnicity and sex, underscoring the need for culturally adapted and stratified Tier 3 intervention to enhance efficacy and equity of service delivery.

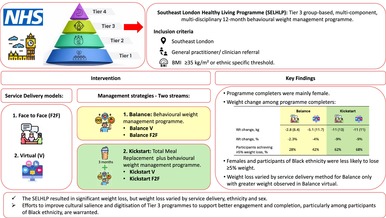

## 1 Introduction

One billion people live with overweight or obesity and prevalence estimates are projected to increase [1, 2] resulting in significant personal [3, 4] and economic [1] costs. Clinical diagnostic criteria for obesity are shifting with new frameworks proposed to guide more targeted prevention and treatment [5, 6]. Pharmacological therapies for obesity management are currently receiving widespread recognition, and their effectiveness for weight loss is undisputed [7]. However, there is concern regarding adverse effects [8], notably loss of skeletal muscle mass and function [9, 10, 11], potential for weight regain and there is a need for longer‐term data [12]. Moreover, not all patients desire [13] or are suitable for [14] GLP‐1 therapy and a 26% discontinuation rate is reported [15]. Access to and affordability [16] of obesity management medication (OMM), while improving, can be challenging and, in the UK, access to OMM can vary [17, 18]. While OMMs result in significant weight loss in both clinical trials [12, 19, 20, 21] and real‐world services [22], there is preliminary data to suggest that behaviour change in addition to weight loss is limited [23, 24]. Behavioural interventions for weight management, which are scalable, can be personalised and tailored to need, have been shown to support behaviour change [25, 26] in addition to modest weight loss [13, 27, 28].

In the UK, Tier 3 weight and behavioural interventions are delivered as part of the National Health Service (NHS) and NICE specifications regarding service design, composition and evaluation exist [29]. However, access to services varies geographically and service delivery and composition can be heterogeneous [27, 30, 31]. While OMMs are increasingly integrated within Tier 3 services [32] the need for behavioural and lifestyle interventions for weight management continues to be recognised by healthcare professionals even in the context of increasing OMM use [13, 17]. However, dropout rates from Tier 3 services are high and engagement of ethnic minorities is limited, despite obesity being higher among ethnically diverse communities [33]. Qualitative data from the UK suggest participants of Black African/Caribbean background are less likely to engage with primary care weight management programmes [34] and lower weight loss is reported among participants that do engage. Dobbie and colleagues reported higher attrition (> 61%) and lower weight change among ethnic minority participants compared to White participants treated with 3 mg liraglutide as part of a Tier 3 service [22].

Access to Tier 3 services is dependent upon funding and delivery across the UK varies. The diversity of services, high dropout rates and suboptimal service evaluations is problematic, and there is a notable absence of data on the impact of these services across different ethnic groups. Additionally, while the link between deprivation and obesity rates is documented [35], there is an absence of data exploring the impact of deprivation on the effectiveness of Tier 3 services.

eHealth delivery of Tier 3 services affords an opportunity to increase the reach and engagement with behavioural and weight management services and provides better patient choice. Evidence for the effectiveness of eHealth Tier 3 services is limited, but data from a digital intervention in Northern England reported comparable weight loss with face‐to‐face (F2F) and digital delivery [36]. The UK NHS Digital Weight Management programme, the first digital intervention with population reach and preliminary data, reports average weight loss was −2.2 kg (95% CI −2.25, −2.16) among 14, 268 participants and greater weight loss was associated with programme completion (−3.9 kg (95% CI −3.99, −3.84)) [37]. Completion rate (45%) of this national digital programme was suboptimal but comparable with existing Tier 3 services [25, 38].

The Southeast London Healthy Living Programme (SELHLP) is a multi‐disciplinary service commissioned across five London boroughs. Two service delivery models, face‐to‐face (F2F) and virtual (V), are used to deliver a group‐based, multicomponent diet and lifestyle interventions to an ethnically diverse population and two programmes are offered. Balance is a diet and lifestyle intervention, and Kickstart uses a total meal replacement approach followed by a diet and lifestyle intervention. This aim of this service evaluation reports on the effectiveness and impact of the SELHLP on weight and behavioural outcomes in an ethnically and socioeconomically diverse UK population.

## 2 Methods

### 2.1 Design

This was a retrospective cohort study conducted as a service evaluation of the South‐East London Healthy Living Programme (SELHLP), a Tier 3 specialist weight management intervention delivered by Guy's and St Thomas' NHS Foundation Trust (GSTT). The evaluation assessed the impact of two structured 12‐month lifestyle interventions, Balance and Kickstart, on weight‐related, clinical and behavioural outcomes. Both programmes were delivered either face‐to‐face (F2F) or virtually (V), and the evaluation followed the SQUIRE reporting guidelines [39] and the National Obesity Observatory Standard Evaluation Framework for Weight Management Interventions [40].

### 2.2 Population

Participants were adults aged 18 years or older with a BMI ≥ 35 kg/m^2^ who enrolled in either the Balance or Kickstart programme. All participants completed baseline assessments with registered dietitians. Outcome data were collected at programme completion. Programme completion was defined as attending one of the final three sessions before August 2020, or one of the final four sessions thereafter, reflecting adaptations during the COVID‐19 pandemic.

### 2.3 Intervention

Balance is a behavioural lifestyle programme focused on dietary improvement, increased physical activity and psychological strategies to support long‐term weight management. Kickstart includes an initial three‐month total diet replacement phase (800–1200 kcal/day), followed by gradual food reintroduction and behavioural lifestyle support. Both interventions were delivered over 12 months via structured group sessions facilitated by a multidisciplinary team including dietitians, psychologists, physicians and other health professionals. Initially, the services offered both programmes in a face‐to‐face (F2F) context but virtual (V) delivery was incorporated due to the COVID‐19 pandemic and retained since.

### 2.4 Outcomes

The primary outcome was change in body weight from baseline to programme completion. Additionally, clinically meaningful weight loss was assessed, defined as ≥ 3%, ≥ 5% and ≥ 10% of initial weight [41, 42]. According to NICE guidelines, effective Tier 3 programmes should achieve at least 5% weight loss in 70% of completers at 12 months. Secondary outcomes included changes in diet quality, eating behaviours, physical activity levels and clinical parameters including HbA1c, blood pressure, lipid profile and patient awareness of their diabetes status. Predictors of programme completion and at least 5% weight loss are also reported.

### 2.5 Data Collection

Baseline data were collected during an initial assessment and included demographic characteristics (age, sex, ethnicity, postcode‐derived deprivation level and relationship status), clinical diagnoses (type 2 diabetes, mental health, hypertension), classification of obesity and behavioural factors (physical activity levels, eating behaviours, motivation and confidence for weight management). Follow‐up exit data were collected at programme completion either in person or online, depending on the service delivery mode.

Weight and BMI were recorded throughout the programme. For the F2F programmes, weight was objectively measured but self‐reported in the virtual programmes. Clinical data including blood pressure, lipid profile (LDL‐C, HDL‐C) and HbA1c were obtained from hospital or surgery laboratory records, with participants encouraged to request repeat bloodwork from their general practitioner (GP) at programme end. Participants were also asked if they were aware of their diabetes status.

Diet quality was assessed using the UK Diabetes and Diet Questionnaire (FFQ), where higher scores indicate poorer diet quality [43]. Binge eating behaviour was assessed using the Questionnaire on Eating and Weight Patterns‐Revised (QEWP‐R) [44], a 27‐item diagnostic tool for binge eating disorder (BED) based on DSM‐IV criteria, also capturing loss of control and distress related to eating. Eating behaviours were further evaluated using the Three‐Factor Eating Questionnaire (TFEQ), which measures cognitive restraint (20 items), disinhibition (16 items) and hunger (15 items) [45].

Physical activity was assessed using the General Physical Activity Questionnaire (GPAQ), which classifies participants into a four‐level Physical Activity Index (PAI): active, moderately active, moderately inactive and inactive [46].

### 2.6 Statistical Analysis

Analyses of weight change and secondary outcomes were restricted to programme completers, whereas baseline characteristics were summarised for all participants.

Descriptive statistics were used to summarise baseline and outcome data. Continuous variables were presented as means with standard deviations and median and interquartile range (IQR). Where appropriate, data presented as median (IQR) in tables, is presented in the text as mean (SD) given it is more clinically familiar. Categorical variables are reported as counts (N) and percentages. Independent *t*‐tests or Mann–Whitney *U* and Kruskal–Wallis tests were used for comparisons of continuous variables, and chi‐squared tests for categorical variables. Between‐group comparisons (e.g., completers vs. non‐completers, face‐to‐face vs. virtual delivery) were performed using the appropriate tests based on variable type and distribution.

Univariable logistic regression was used to examine potential predictors of programme completion and achievement of ≥ 5% weight loss. Candidate predictors included demographic variables (age, sex, ethnicity, deprivation level, relationship status). Variables with *p* < 0.05 in univariable analysis were included in multivariable logistic regression models using forced entry. Results were reported as odds ratios (OR) with 95% confidence intervals (CI). All analyses were conducted in IBM SPSS Statistics version 29, and *p* < 0.05 was considered statistically significant.

### 2.7 Ethical Considerations

This project was conducted as a quality improvement service evaluation using anonymised data and did not require NHS ethical approval. Approval for data use and analysis was obtained from the Quality Improvement and Patient Safety committee at GSTT (Reference 14211).

## 3 Results

### 3.1 Participant Characteristics

Overall, the SELHLP cohort was predominantly female (79%), 47% of participants were of White ethnicity and 37% reported Black ethnicity. Programme completion, reported elsewhere, was 43% (Balance 43.5%; Kickstart 42.6%) (O'Keeffe et al. unpublished).

Table 1 outlines participant characteristics of programme completers compared to non‐completers by Balance or Kickstart programme (F2F and V combined). Participant characteristics were relatively similar across programmes. Participants that completed Kickstart were more likely to be female (*p* = 0.007), older (*p* = 0.015) and marginally more deprived (*p* < 0.001) compared to non‐completers (Table 1). Programme completion rates differed by ethnicity for Balance and by sex for Kickstart (Table 1). Participants of Black ethnicity were less likely to complete Balance (completer 32% vs. 39% non‐completer, *p* = 0.015) whereas men were less likely to complete Kickstart (completer 21% vs. non‐completer 29%, *p* = 0.007).

**TABLE 1:** Participant characteristics for programme completers and non‐completers of the Southeast London Healthy Living Healthy Programme.

	Balance^a^			Kickstart^b^		
	Completer	Non‐completer	*p*	Completer	Non‐completer	*p*
	*n* (%)	*n* (%)		*n* (%)	*n* (%)	
Sex, *n*	565	730	*χ* ^2^ = 2.153, df(1), *p* = 0.142	324	436	*χ* ^2^ = 7.34, df(1), *p* = 0.007
Male	100 (18)	153 (21)		67 (21)	128 (29)	
Female	465 (82)	577 (79)		257 (79)	308 (71)	
Ethnicity, *n*	549	718	*χ* ^2^ = 8.427, df(2), *p* = 0.015	321	430	*χ* ^2^ = 0.772, df(2), *p* = 0.68
White	275 (50)	343 (48)		170 (53)	217 (51)	
Black African/Caribbean	176 (32)	279 (39)		108 (34)	158 (37)	
Other	98 (18)	96 (13)		43 (13)	55 (13)	
Relationship status, *n*	378	504	*χ* ^2^ = 2.647, df(2), *p* = 0.266	292	427	*χ* ^2^ = 2.174, df(2), *p* = 0.337
Single	189 (50)	226 (45)		120 (41)	160 (42)	
Married/civil partner	134 (35)	190 (38)		136 (47)	160 (42)	
Separated/divorced/widowed	55 (15)	88 (18)		36 (12)	60 (16)	
Living arrangements, *n*	375	718	*χ* ^2^ = 3.581, df(6), *p* = 0.733	292	383	*χ* ^2^ = 6.831, df(6), *p* = 0.337
Alone	84 (22)	118 (23)		64 (22)	65 (17)	
Spouse/partner	75 (20)	107 (21)		67 (23)	79 (21)	
Children	97 (26)	127 (25)		59 (20)	92 (24)	
Parents/relatives	42 (11)	56 (11)		21 (7)	36 (9)	
Roommates/friends	13 (4)	9 (2)		5 (2)	11 (3)	
Spouse/partner and children	62 (17)	89 (18)		75 (26)	100 (26)	
Prefer not to say	2 (0.5)	1 (0.2)		1 (0.3)	—	

	*N*	Mean (SD)	*N*	Mean (SD)	*p*	*N*	Mean (SD)	*N*	Mean (SD)	*p*
Age, years	566	48.5 (12.7)	724	48.5 (14.6)	*Z* = −0.616, *p* = 0.538	323	48.8 (12.2)	436	46.6 (12.7)	*Z* = −2.43, *p* = 0.015
IMD, decile	546	4 (2.3)	704	4 (2.4)	*Z* = 0.658, *p* = 0.511	314	5 (2.6)	416	5 (2.5)	*Z* = −3.486, *p* < 0.001
Income, decile	546	4 (2.4)	704	4 (2.5)	*Z* = −0.274, *p* = 0.784	314	5 (2.7)	416	4 (2.6)	*Z* = −2.765, *p* = 0.005
Employment, decile	546	5 (2.4)	704	5 (2.5)	*Z* = −0.144, *p* = 0.886	314	5 (2.7)	416	5 (2.5)	*Z* = −3.095, *p* = 0.002
Education and skills, decile	546	6 (2)	704	6 (2.1)	*Z* = −0.693, *p* = 0.489	314	7 (2.2)	416	6 (2.2)	*Z* = −2.873, *p* = 0.004
Health and disability, decile	546	5 (2.3)	704	6 (2.5)	*Z* = −1.579, *p* = 0.115	314	6 (2.5)	416	6 (2.5)	*Z* = −3.274, *p* = 0.001
Crime, decile	546	4 (2.1)	704	4 (2.2)	*Z* = −1.723, *p* = 0.085	314	5 (2.3)	416	4 (2.2)	*Z* = −2.558, *p* = 0.011
Barriers to housing and services, decile	546	3 (1.5)	704	3 (1.5)	*Z* = −0.592, *p* = 0.554	314	3 (1.7)	416	3 (1.7)	*Z* = −2.19, *p* = 011
Living environment, decile	546	3 (1.8)	704	4 (1.9)	*Z* = −2.655, *p* = 0.008	314	4 (2.1)	416	4 (2)	*Z* = −1.678, *p* = 0.093

*Note:* Data is presented for the combined cohorts for each programme stream, Balance (face‐to‐face and virtual) and Kickstart (face‐to‐face and virtual). Data as *n* (%) or means ± SD. Chi‐squared test for association and Mann–Whitney *U* tests. Statistical significance considered at *p* < 0.05.

^a^ Balance includes Balance face‐to‐face, Balance virtual.

^b^ Kickstart includes Kickstart face‐to‐face, Kickstart virtual.

### 3.2 Clinical Status and Anthropometry

Overall, the cohort for both programmes was hypertensive, 36% had type 2 diabetes as determined by HbA1c, and depression and anxiety were common. 85% of the cohort were living with class III obesity. There was no difference in clinical status between completers and non‐completers of Balance, but for Kickstart, more people with type 2 diabetes (*p* = 0.007) and depression (*p* = 0.005) did not complete Kickstart (Table 2). Weight at baseline was similar across programmes and did not impact programme completion (Table 2).

**TABLE 2:** Anthropometry and clinical status of completers and non‐completers by programme stream.

	Balance^a^					Kickstart^b^				
	Completer		Non‐completer		*p*	Completer		Non‐completer		*p*
	*N*	Mean (SD)	*N*	Mean (SD)		*N*	Mean (SD)	*N*	Mean (SD)	
Weight, kg	562	124.2 (19.7)	718	123.2 (20.6)	*Z* = −0.946 *p* = 0.344	323	121.5 (18.5)	428	125.6 (23)	*Z* = −1.673 *p* = 0.094
BMI, kg/m^2^	551	45.1 (6)	686	44.6 (6)	*Z* = −1.598 *p* = 0.11	314	44.2 (6)	405	44.4 (6.2)	*Z* = −0.439 *p* = 0.661
Blood pressure										
SBP, mmHg	513	132 (15)	610	133 (16)	*Z* = −1.112 *p* = 0.266	299	133 (15)	360	134 (16)	*Z* = −0.806 *p* = 0.42
DBP, mmHg	511	82 (9)	609	83 (11)	*Z* = −0.787 *p* = 0.431	299	82 (10)	360	84 (9)	*Z* = −1.868 *p* = 0.062
Lipids										
Total cholesterol, mmol/L	449	4.7 (1)	401	4.7 (1)	*Z* = −0.829 *p* = 0.407	208	4.9 (3.3)	262	4.7 (1.1)	*Z* = −0.815 *p* = 0.415
HDL‐c, mmol/L	408	1.4 (0.5)	361	1.3 (0.4)	*Z* = −1.612 *p* = 0.107	238	1.4 (0.6)	240	1.3 (0.5)	*Z* = −1.825 *p* = 0.068
LDL‐c, mmol/L	364	2.6 (1)	313	2.6 (1)	*Z* = 0.699 *p* = 0.485	261	2.5 (1)	198	2.7 (1)	*Z* = −1.777 *p* = 0.076
HbA1c, mmol/mol	472	47 (14.7)	425	49 (16.5)	*Z* = −1.123 *p* = 0.261	276	46.9 (16.2)	271	49 (16)	*Z* = −1.315 *p* = 0.188

	*N* (%)	*N* (%)	*p*	*N* (%)	*N* (%)	*p*
Diabetes status by HbA1c	467	418	*χ* ^2^ = 1.938, df(2), *p* = 0.379	273	269	*χ* ^2^ = 5.323, df(2), *p* = 0.007
Normal, ≤ 41 mmol/mol	215 (46)	173 (41)		137 (50)	123 (46)	
Prediabetes, 42–47 mmol/mol	100 (18)	97 (23)		59 (22)	46 (17)	
Diabetes, ≥ 48 mmol/mol	152 (33)	148 (35)		77 (28)	100 (37)	
Mental health diagnosis	499	639	*χ* ^2^ = 4.775, df(3), *p* = 0.189	299	396	*χ* ^2^ = 12.826, df(3), *p* = 0.005
Depression	86 (17)	130 (20)		43 (14)	90 (23)	
Anxiety/Anxiety & depression	48 (10)	73 (11)		30 (10)	28 (7)	
Other	25 (5)	21 (3)		10 (3)	4 (1)	
No mental health condition	340 (68)	415 (65)		216 (72)	274 (69)	

*Note:* Data is presented for the combined cohorts for Balance (face‐to‐face and virtual) and Kickstart (face‐to‐face and virtual). Other mental health diagnoses include anxiety, trauma/post‐traumatic stress disorder, bereavement and other conditions. Data as *n* (%) or means ± SD. Statistical significance considered at *p* < 0.05. Chi‐squared test for association and Mann–Whitney *U* tests.

^a^ Balance includes Balance face‐to‐face, Balance virtual.

^b^ Kickstart includes Kickstart face to face, Kickstart virtual.

### 3.3 Impact of Balance and Kickstart on Weight Among Programme Completers

For Balance, among the 471 participants included in the matched analysis, overall weight loss was significant among programme completers (change from baseline, −3.8 [10] kg, *Z* = −8.79, *p* < 0.001). Greater weight loss was observed in the Balance V (−5.1 (11.7 kg) programme compared to Balance F2F −2.8 (8.4) kg; Table 3).

**TABLE 3:** Weight and body mass index change for programme completers of the Tier 3 healthy weight programme.

	*N*	Baseline	End of programme	*p* ^b^
Balance^a^				
Weight, kg (median, IQR)	471	122 (25)	118.8 (27)	*Z* = −8.79, *p* < 0.001
Weight change, kg (median, IQR)			−3 (9.1)	
Weight change, % (median, IQR)			−2.4	
BMI, kg/m^2^ (median, IQR)	461	43.9 (6.5)	42.6 (8.1)	*Z* = −8.964, *p* < 0.001
	Mean (SD)			
Weight, kg	471	124 (19.6)	121.2 (21.3)	
Weight change, kg			−3.8 (9.3)	
Weight change, %			−3	
BMI, kg/m^2^	461	45 (6)	43.6 (6.8)	
Balance F2F				
Weight, kg (median, IQR)	275	123 (26)	119.7 (28.4)	*Z* = −5.757, *p* < 0.001
Weight change, kg (median, IQR)			−2.2 (8)	
Weight change, % (median, IQR)			−2	
BMI, kg/m^2^ (median, IQR)	271	43.9 (6.3)	42.8 (7.5)	*Z* = −6.039, *p* < 0.001
	Mean (SD)			
Weight, kg	275	124.2 (19.7)	121.4 (21.4)	
Weight change, kg			−2.8 (8.4)	
Weight change, %			−2.3	
BMI, kg/m^2^	271	44.9 (5.8)	43.8 (6.7)	
Balance V				
Weight, kg (median, IQR)		120.6 (23.5)	116.8 (24.1)	*Z* = −6.664, *p* < 0.001
Weight change, kg (median, IQR)			−4.9 (11.8)	
Weight change, % (median, IQR)			−4	
BMI, kg/m^2^	191	45.2 (6.2)	43.3 (7.1)	
	Mean (SD)			
Weight, kg	196	123.6 (19.4)	118.5 (21.1)	
Weight change, kg			−5.1 (11.7)	
Weight change, %			−4	
BMI, kg/m^2^ (median, IQR)		43.9 (7.1)	41.9 (8.7)	*Z* = −6.534, p < 0.001
Kickstart^a^				
Weight, kg (median, IQR)		119.5 (26)	109 (26.8)	*Z* = −11.953, *p* < 0.001
Weight change, kg (median, IQR)			−9.5 (15)	
Weight change, % (median, IQR)			−8	
BMI, kg/m^2^ (median, IQR)		42.8	40	*Z* = −11.48, *p* < 0.001
	Mean (SD)			
Weight, kg	268	121.5 (18.7)	110.5 (20.2)	
Weight change, kg			−11 (13)	
Weight change, %			−9	
BMI, kg/m^2^	261	44.1 (5.5)	40.3 (6.5)	
Kickstart F2F				
Weight, kg (median, IQR)		118 (25.7)	108.9 (25.9)	*Z* = −8.851, *p* < 0.001
Weight change, kg (median, IQR)			−10 (15.2)	
Weight change, % (median, IQR)			−8.3	
BMI, kg/m^2^ (median, IQR)	136	42.7 (6.4)	40 (6.8)	*Z* = −8.636, *p* < 0.001
	Mean (SD)			
Weight, kg	140	119.7 (18.1)	108.7 (18.4)	
Weight change, kg			−11 (13)	
Weight change, %			−9	
BMI, kg/m^2^	136	43.9 (5.2)	40 (6)	
Kickstart V				
Weight, kg (median, IQR)		121 (24.3)	109.2 (29.3)	*Z* = −8.043, *p* < 0.001
Weight change, kg (median, IQR)			−9 (13.5)	
Weight change, % (median, IQR)			−7.5	
BMI, kg/m^2^ (median, IQR)	123	44 (7.8)	40 (9)	*Z* = −7.608, *p* < 0.001
	Mean (SD)			
Weight, kg	128	123.5 (16.3)	112.5 (21.9)	
Weight change, kg			−11 (11)	
Weight change, %			−9	
BMI, kg/m^2^	123	44.4 (5.7)	40.7 (7)	

*Note:* Data is given at baseline and end of programme (12 months). Data is presented as median (IQR) and means (SD) unless otherwise indicated. Statistical significance considered at *p* < 0.05.

^a^ Face to face and virtual programmes combined.

^b^ Wilcoxon signed rank test, baseline to 12 months.

73% of completers of Balance V and 67% of completers of Balance F2F lost or maintained their weight, and the average weight change among these participants was −10 (8.8) kg and−7 (6.6) kg for Balance V and F2F respectively (Table 4).

**TABLE 4:** Percentage of programme completers that lost or maintained weight across the different Tier 3 programme streams.

	*n*/*N*	%	Weight change, kg	Weight change, %	Weight change range, kg, max–min	Weight change range, %, min–max
Balance^a^	325/472	69	−8.1 (7.8)	−6.5 (6)	−51, 0	−31.2, 0
Balance F2F	182/276	67	−7 (6.6)	−5.5 (5)	−51, 0	−29, 0
Balance V	143/196	73	−10 (8.8)	−7.7 (6.7)	−38, 0	−31.2, 0
Kickstart^b^	229/269	85	−14 (11.2)	−11.2 (8.3)	−63.2, 0	−41.7, 0
Kickstart F2F	119/141	84	−14 (11)	−11.4 (8.2)	−63.2, −0.4	−41.7, −0.4
Kickstart V	110/128	86	−14 (11.5)	−11.1 (8.4)	−53.8, 0	−35.7, 0

*Note:* The weight profile (kg, %) of this cohort is also presented including the range of weight change.

^a^ Balance includes Balance face‐to‐face, Balance virtual.

^b^ Kickstart includes Kickstart face‐to‐face, Kickstart virtual.

Average weight loss among completers of Kickstart was −11 (13) kg and there was no difference in weight change between programme delivery methods (Kickstart F2F −11 (12.5 kg) vs. Kickstart V 11 (11) kg, *p* > 0.05). The proportion of people losing or maintaining weight was similar for the virtual versus F2F Kickstart programme (86% and 84% respectively) and the degree of weight loss among these participants was also similar (14 (11) kg vs. 14 (11.5) kg for F2F vs. V Kickstart) (Table 4).

**FIGURE 1:**
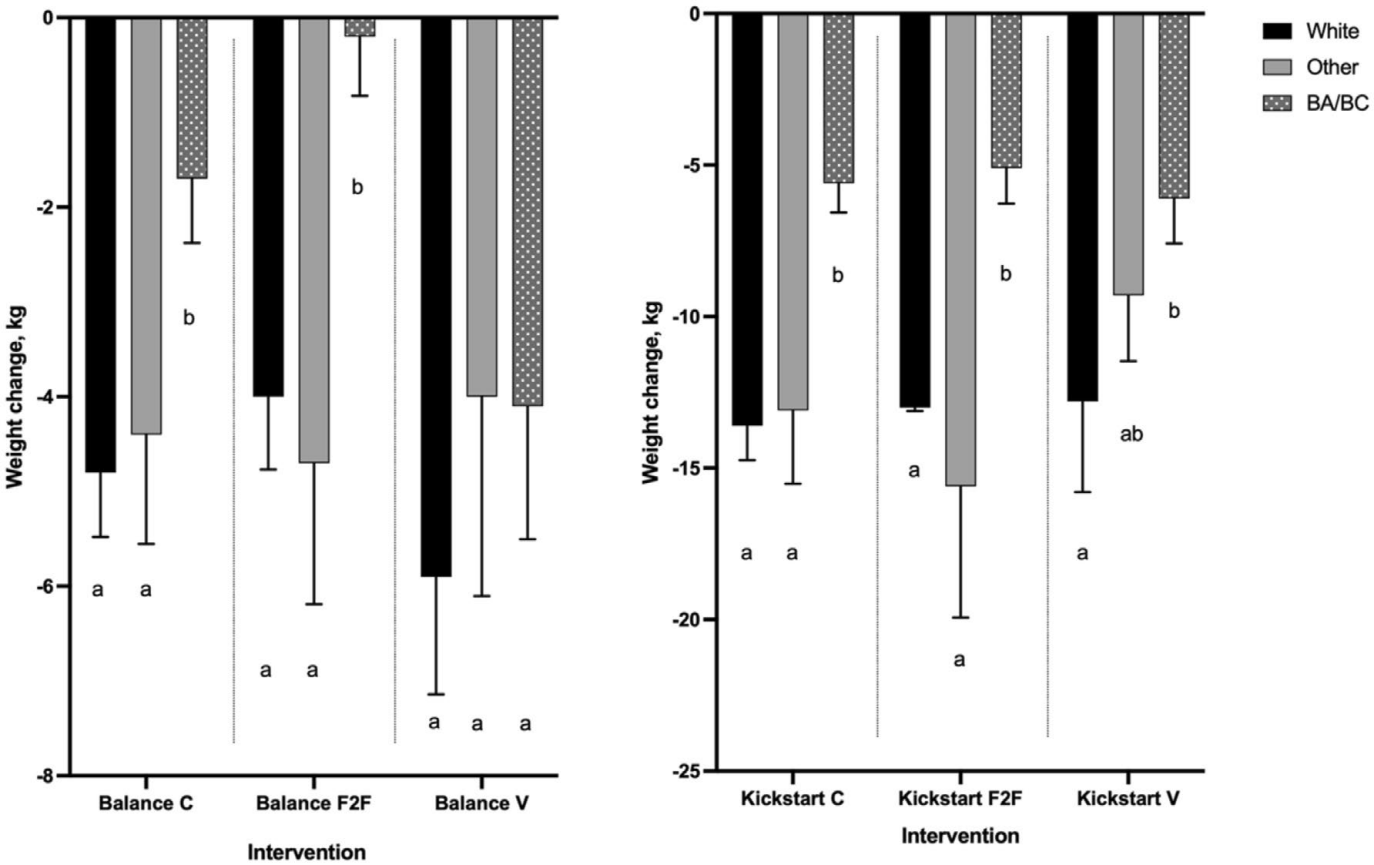
Weight loss (kg) by programme and ethnicity for completers of both Balance and Kickstart. Data is reported as mean (SEM) and statistical significance is considered at *p* ≤ 0.05. BA/BC, Black African/Black Caribbean ethnicity; C, combined; F2F, face‐to‐face; V, virtual. a, *p* < 0.05; b, *p* > 0.05.

The impact of Balance and Kickstart on weight varied by ethnicity (Figure 1; Table S1). Across all programmes, apart from Balance virtual, participants of Black African/Caribbean ethnicity lost less weight. Balance F2F had the worst outcomes with a recorded weight loss of −0.2 kg [6] recorded for those of Black ethnicity compared to −4 (8.9) kg and −4.7 (10.1) kg for participants of White and Other ethnic background respectively. However, for the virtual Balance programme there was no difference in weight loss across ethnic groups (*p* = 0.2).

### 3.4 Weight Loss Targets: 3%, 5% and 10% Weight Loss

Table 5 outlines clinically meaningful, 3%, 5% and 10% weight loss among programme completers by programme stream. 40% of participants that completed Balance F2F lost ≥ 3% of initial weight and the average weight change was −8.1 (4.7)%. However, 56% of those that completed Balance V lost ≥ 3% with the average weight change of −9.8 (6.3)%. Over 70% of Kickstart completers achieved ≥ 3% weight loss and the average weight loss was −12.7 [8] % and −13.1 (7.8)% for Kickstart F2F and V respectively. For the combined cohorts (F2F and V), 34% of Balance completers and 64% of Kickstart completers lost ≥ 5% of weight; average percent weight change for Balance was 10.7 (5.6)% and 14.2 (7.5)% for Kickstart. The percentage of those achieving ≥ 10% weight loss was 14% for Balance combined and 41% for Kickstart combined, a weight change of −15.7 (5.5)% and −18 (7)% respectively (Table 4).

**TABLE 5:** Programme completers that lost 3%, 5% and 10% of weight at programme end.

	*n*/*N*	%	Weight change, kg	Weight change, %
≥ 3% weight loss				
Balance^a^	219/471	47	−11.3 (7.7)	−9 (5.6)
Balance F2F	110/276	40	−10.1 (6.6)	−8.1 (4.7)
Balance V	109/196	56	−12.4 (8.4)	−9.8 (6.3)
Kickstart^b^	197/269	73	−16 (11)	−13 (7.8)
Kickstart F2F	105/141	75	−15.4 (10.6)	−12.7 (8)
Kickstart V	92/128	72	−16.4 (11.1)	−13.1 (7.8)
≥ 5% weight loss				
Balance^a^	160/471	34	−13.6 (7.8)	−10.7 (5.6)
Balance F2F	78/276	28	−12.2 (7)	−9.7 (4.6)
Balance V	82/196	42	−15 (8.4)	−11.7 (6.2)
Kickstart^b^	171/269	64	−17.6 (10.6)	−14.2 (7.5)
Kickstart F2F	87/141	62	−17.8 (10.3)	−14.5 (7.5)
Kickstart V	84/128	66	−17.5 (11)	−14 (7.6)
≥ 10% weight loss				
Balance^a^	67/471	14	−20 (8.1)	−15.7 (5.5)
Balance F2F	31/276	11	−17.8 (7.7)	−14.1 (4.4)
Balance V	36/196	18	−22 (8.2)	−17.1 (6)
Kickstart^b^	111/269	41	−22.2 (10.5)	−18 (7)
Kickstart F2F	62/141	44	−21.2 (10.3)	−17.2 (7.1)
Kickstart V	49/128	38	−23.5 (10.8)	−18.7 (6.6)

*Note:* Weight change (kg, %) among these participants are also reported.

^a^ Balance includes Balance face‐to‐face, Balance virtual.

^b^ Kickstart includes Kickstart face‐to‐face, Kickstart virtual.

### 3.5 Predictors of ≥ 5% Weight Loss

In the univariable models for the Balance programme, age, sex and ethnicity were significant predictors of ≥ 5% weight loss (Table S2). For age (OR 1.017, 95% CI 1.002–1.032, *p* = 0.029), older participants had greater odds of achieving the weight loss target and female participants were half as likely to achieve ≥ 5% weight loss compared to males (OR 0.488, 95% CI 0.301–0.790, *p* = 0.004). Regarding ethnicity, participants of Black African/Caribbean ethnicity had almost 60% lower odds of achieving this weight loss target compared to White participants (OR 0.402, 95% CI 0.259–0.624, *p* < 0.001). Low deprivation compared to high deprivation levels was found to be predictive of ≥ 5% weight loss (OR 1.573, 95% CI 1.000–2.475, *p* = 0.050). There was a non‐significant trend for married/civil partnered to have marginally better outcomes compared (OR 1.593, 95% CI 0.957–2.652, *p* = 0.073). Weight at baseline had no impact (OR 1.006, 95% CI 0.996–1.015, *p* = 0.26).

In the multivariable regression (Table 6), only sex and ethnicity remained significant predictors. Females were 44% less likely to achieve ≥ 5% weight loss compared to males (OR 0.563, 95% CI 0.332–0.956, *p* = 0.033). Participants of Black African/Caribbean ethnicity were 46% less likely to achieve the weight loss goal (OR 0.541, 95% CI 0.337–0.869, *p* = 0.011). The effect of deprivation and age was lost suggesting that the effect of these was confounded by ethnicity and sex.

**TABLE 6:** Multivariable binary regression models for predictors of ≥ 5% weight loss for Balance (*n* = 455) and Kickstart (*n* = 255).

	Balance combined					Kickstart combined				
	Β (SE)	Wald	df	OR (95% CI)	*p*	Β (SE)	Wald	df	OR (95% CI)	*p*
Sex										
Male (ref)	—	—	—	1		—	—	—	1	
Female	−0.574 (0.27)	4.527	1	0.563 (0.332–0.956)	0.033	−1.483 (0.473)	9.819	1	0.227 (0.09–0.574)	0.002
Ethnicity										
White (ref)	—	—	—	1		—	—	—	1	
Black	−0.614 (0.242)	6.446	1	0.541 (0.337–0.869)	0.011	−0.731 (0.313)	5.453	1	0.481 (0.261–0.889)	0.02
Other	−0.233 (0.246)	1.014	1	1.281 (0.791–2.073)	0.314	0.557 (0.45)	1.534	1	1.745 (0.723–4.212)	0.216
Deprivation										
High	—	—	—	1		—	—	—	1	
Moderate	0.171 (0.245)	0.487	1	1.186 (0.734–1.917)	0.485	0.089 (0.365)	0.059	1	1.093 (0.534–2.234)	0.808
Low	0.247 (0.246)	1.014	1	1.281 (0.791–2.073)	0.314	0.225 (0.336)	0.449	1	1.253 (0.648–2.422)	0.503
Age	0.013 (0.008)	2.785	1	1.013 (0.988–1.029)	0.095	0.026 (0.012)	4.559	1	1.026 (1.002–1.05)	0.033

*Note:* Balance: Cox & Snell *R* ^2^ = 0.052; Nagelkerke *R* ^2^ = 0.07. Kickstart: Cox & Snell *R* ^2^ = 0.135; Nagelkerke *R* ^2^ = 0.185.

For Kickstart, in univariable analyses, age, sex and ethnicity were significant predictors of ≥ 5% weight loss (Table S2). Similar to Balance, a non‐significant trend for achieving ≥ 5% weight loss was identified for participants that were married/civil partnered or those with low levels of deprivation was also observed (Table S2).

In the multivariable analysis for Kickstart, women were 84% less likely to achieve the ≥ 5% weight loss target (*p* = 0.02) (Table 6). The effect of ethnicity was maintained; those of Black ethnic origin were 63% less likely (OR 0.541, 95% CI 0.377, 0.869, *p* = 0.02) to reach ≥ 5% weight loss, and older adults were more likely to achieve ≥ 5% weight loss (*p* = 0.033). The effect of age was stronger in Kickstart compared to Balance (Table 6). The effect of deprivation seen in the univariable model (Table S2) was lost in the multivariate multivariable analyses (Table 6), suggesting that sex and ethnicity were confounders, mirroring what was observed for Balance.

### 3.6 Behavioural and Clinical Outcomes

#### 3.6.1 Diet Quality and Eating Behaviour

Among programme completers, dietary quality improved by 6% for Balance (*Z* = −3.081, *p* = 0.002) and 12.5% for Kickstart (−2.179, *p* = 0.029) (Table S3). There was a small but statistically significant worsening of binge eating (*Z* = −2.781, *p* = 0.005 Balance; *Z* = −3.254, *p* = 0.001 Kickstart), as quantified by the QEWP‐R, in both programmes but no effect on eating behaviours as categorised by the TEFQ (Table S3).

#### 3.6.2 Physical Activity

Physical activity data at both baseline and follow‐up were available for only 128 participants (22.6%) of Balance. At baseline, 32% were classified as inactive and 30% as moderately inactive. At follow‐up, the proportion classified as inactive remained similar (31%) while the proportion classified as moderately inactive decreased to 18%, with corresponding increases in the moderately active (16%–22%) and active (22%–29%) categories. Baseline physical activity category was significantly associated with follow‐up category (*χ* ^2^ = 53.753, df = 9, *p* < 0.001), with a significant linear trend confirming that higher baseline activity predicted higher follow‐up activity (linear‐by‐linear association 29.799 df(1) *p* < 0.001; Table S3).

A similar trend was observed for physical exercise duration; at baseline, 77% of participants reported doing no weekly structured physical exercise, and only 7% reported > 3 h per week. At follow‐up, 58% reported no weekly exercise at follow‐up, 14% reported > 1 h, 20% reported 1–3 h and 7.5% reported > 3 h per week. Participants that did not exercise at baseline were most likely to remain in this category. Similar to Balance, there was a linear trend for the physical activity index for Kickstart (linear‐by‐linear association 11.861 df(1) *p* < 0.001) but no overall association between baseline and end of programme (*p* = 0.053). The missingness of the Kickstart data was high, with only matched data available for the physical index available for 63 participants. Duration of physical exercise (*p* = 0.052) and walking remained unchanged (*p* = 0.273; Table S3).

#### 3.6.3 Lipids, Blood Pressure and Diabetes Status

A small but non‐significant reduction in LDL‐C (*p* = 0.079) and blood pressure (SBP, *p* = 0.079; DBP, *p* = 0.067) was observed in the combined Balance cohort, whereas improvement in both LDL‐C and HDL‐C were observed with combined Kickstart cohort (Table S2). Despite greater reductions in weight compared to the Balance programme, HbA1c increased by 7.6 mmol/mol (18%) in the combined Kickstart programme (Table S2). A sensitivity analysis on the impact of both programmes on HbA1c among programme completers with matched HbA1c data was undertaken. For the combined Balance cohort (*n* = 173), a reduction in both weight (BL 120 (24.7) kg versus EOP 113.5 (26) kg, Z = −5.879, *p* < 0.001) and HbA1c was observed (BL 45 (17.5) mmol/mol versus EOP 44 (15) mmol/mol, *Z* = −2.645, *p* = 0.008). However, for Kickstart (*n* = 66), there was only a reduction in weight (BL 115 (25) kg vs. EOP 103.3 (24) kg, *Z* = −6.222, *p* < 0.001) but no effect on HbA1c (BL 45 (11) mmol/mol vs. EOP 44 (16) mmol/mol, *Z* = −0.12, *p* = 0.904).

## 4 Discussion

The SELHLP had a significant impact on weight and behavioural outcomes, independent of service delivery model, ethnicity or deprivation status. Both Balance and Kickstart resulted in an average weight loss of −3.8 (10) kg and −11 (13) kg respectively. No difference in weight loss was observed between the virtual and face‐to‐face programmes for participants on the TMR‐based programme, Kickstart. However, for participants on the diet and lifestyle intervention, Balance, virtual delivery resulted in twice as much weight loss compared to the face‐to‐face programme. For participants that lost ≥ 5% of initial weight, significant weight loss was recorded independent of the intervention type or service delivery method. Participants of Black African/Caribbean ethnicity experience less weight loss in all programmes, apart from Balance Virtual where there was no difference in weight loss by ethnicity.

Both the diet and lifestyle intervention and the TMR resulted in clinically significant weight change among participants of both programmes. 42% of completers of Balance V and 28% of Balance F2F lost ≥ 5% of initial weight by 12 months, meaning the primary outcome of 70% of completers losing ≥ 5% of initial weight was not met. However, this is consistent with other Tier 3 programmes. In the primary‐care Fakenham weight management service, 53.8% of completers lost 5% or more [25], whereas in the Aintree LOSS community‐based multidisciplinary service, 24.1% of participants achieved this target [47]. In Northern England, 54% of participants who completed a 12‐week Tier 3 programme lost weight, but only 16% lost five or more percent [48]. For Kickstart, 62% and 66% of completers of the F2F and virtual programmes achieved the 5% weight loss target, which again did not meet the 70% outcome. In Scotland, Lean and colleagues reported that a low‐energy liquid diet formula with food reintroduction resulted in an average weight loss of −12.4 (11.4) kg or −9.1% weight loss, which is comparable to the weight loss observed with combined Kickstart programme −11 (13) kg or −9% [49]. 56% of participants on the Counterweight Programme, which used TMR intervention, completed the programme and of these, 40% had an average weight loss of 14.2 kg and 28% of participants lost > 10% weight [50], which is consistent with our findings from Kickstart.

Negative predictors of at least 5% weight loss for both Balance and Kickstart included being female and of Black ethnicity, while older age was a positive predictor. However, in the Balance multi‐variable analysis only ethnicity and sex persisted as a predictors; Black participants were 46% less likely to reach at least 5% weight loss. A similar pattern was observed for the multivariable analyses for Kickstart. Being of Black descent and female were 52% and 77%, respectively, less likely to achieve ≥ 5% weight loss. The effect of older age was more pronounced in Kickstart compared to Balance. The impact of age on weight loss is mixed; in the POUNDS study, a two‐year clinical trial among majority White and female participants, age and race were positive predictors of weight loss [51], but other studies do not report age as a significant predictor of weight loss [52, 53, 54, 55].

The lower likelihood of weight loss with diet and lifestyle intervention among participants of Black ethnicity has been previously reported [56] however UK specific data is sparse. In one of the only studies to focus on people of Black ethnicity in the UK, Maynard and colleagues undertook a qualitative investigation of the lived experience of Black African and Black Caribbean adults and their perspectives of weight management services [34]. Experienced or anticipated racism from healthcare services, a contested view of the role of primary care in weight management and lack of cultural saliency of these interventions were cited as barriers. In our cohort, 36% of the overall population were of Black origin which suggests good engagement with the SELHLP. However, Black ethnicity was associated with lower completion rates (O'Keeffe et al. unpublished) and weight loss suggesting that efforts to enhance the cultural relevance of the programme are warranted and this is consistent with the literature. In the US, Black African Americans are often underrepresented in weight loss programmes and tend to lose less weight than White participants [57, 58, 59]. However, ethnic representation within [56] and cultural tailoring of interventions [60, 61] can improve the effectiveness of diet and behavioural interventions for obesity treatment. Our study is the first to report lower completion rates (O'Keeffe et al. unpublished) and lower likelihood of weight loss among participants of Black African and Black Caribbean origin in the UK and our findings directly respond to calls for such programmes evaluations to consider differences by age, ethnicity, sex and socioeconomic status [34]. Furthermore, the findings warrant a review of existing NHS Tier 3 services to ensure cultural acceptability and relevance among these participants and strategies to address, not only early engagement, but programme completion. Efforts to tailor NHS services may enable better health outcomes in the context of existing health and social inequalities and an evaluation of the tailored UP!UP! programme in London will be a welcomed addition to the evidence [62].

In a pooled analysis of two, 12‐week weight loss interventions, Batterham and colleagues make a compelling argument for different interventional approaches to weight management and outline how baseline demographic characteristics can impact the trajectory of weight change [63]. Based on our data, a better understanding of the variables that predict weight loss could inform triaging of patients to specific interventions or delivery models that may result in greater weight loss and improved obesity‐related outcomes as well as programme completion rates.

Both programmes resulted in small improvements in clinical and behavioural outcomes but varied by intervention type. Both programmes also resulted in small but significant improvements in dietary quality and physical inactivity. However, Kickstart had no impact on physical activity or exercise, but the data is limited by small sample sizes. A notable improvement in fruit and vegetable intake, a proxy for dietary quality, was reported in Fakenham Tier 3 weight management service [25] and similarly, eating behaviours have also been shown to improve with Tier 3 interventions [26].

LDL‐cholesterol was reduced with both programmes, and Balance also incurred a reduction in blood pressure, albeit non‐significantly. Referral data, which is not presented as it was not part of the service evaluation, indicated that polypharmacy was significant among participants with the majority of participants prescribed antihypertensives and statins which may have masked the impact of weight changes on clinical parameters. HbA1c was unaffected by the Balance intervention but, unexpectedly, increased by 17.8% with Kickstart. Given the degree of weight loss observed with the Kickstart intervention, 11 kg (9%) the increase in HbA1c is unexplained and contrasts with the results of the DROPLET Trial [64] where improvements in both HbA1c and diastolic blood pressure were reported with a TMR intervention. The NHS Type 2 Diabetes Path to Remission Programme has relevance to our finding [65]. Participants who completed the programme and for whom two HbA1c measurements were available, average weight loss was 14.4% but type 2 diabetes in remission was only 32%. Whilst the definition of remission was robust, the data indicates that whilst substantial weight loss occurred with a total meal replacement intervention changes in HbA1c were not observed for all participants. Our finding of increased HbA1c is perplexing and with 9% weight loss we anticipated a modest reduction. It is possible that medication de‐escalation during rapid weight loss or a rebound in carbohydrate intake during food reintroduction may have contributed to the observed increase in HbA1c. However, these remain speculative explanations, as no published evidence currently confirms such mechanisms. Additionally, the translation of research findings into a real world context may demand greater power and further research is needed.

The SELHLP was originally designed as an in‐person, community and group‐based Tier 3 service. However, the COVID‐19 pandemic forced the service to create an eHealth model and both Balance and Kickstart virtual were rolled out to address local needs. The virtual programmes also increased reach and accessibility and, in fact, for Balance virtual, greater weight loss was observed compared to the face‐to‐face service. Absolute weight loss from Balance virtual programmes aligns with the reported weight loss from the Way to Wellness programmes in Wakefield, UK, −5.1 kg versus −6.1 kg [36] and similarly the national prospective data from the NHS digital weight [37]. Interestingly, the effectiveness of the Kickstart intervention was independent of service delivery method, and equal weight loss was observed.

Upon reinstatement of the face‐to‐face programmes following the pandemic, the virtual options were preserved in the service delivery model offering increased options to eligible patients. The diversity of service delivery (virtual or face‐to‐face) and intervention type (diet and lifestyle or total meal replacement plus diet and lifestyle) is a strength of the current service. The multicomponent, multidisciplinary intervention that was developed in line with NICE recommendations for Tier 3 services is another strength of the service. Nationally [66] and internationally [67], efforts to deliver care in communities are recommended and the broad geographic spread of the SELHLP reflects this policy context. Moreover, the service integrates community health service with primary care delivering on the NHS Long Term Plan [66]. Lastly, this service evaluation was undertaken independently of the clinical team who are responsible for service delivery and data collection.

Several limitations should also be considered. Collation of clinical data, notably lipids, blood pressure and HbA1c, was very challenging as these had to be provided by participants' general practitioner so access to the data was limited and impacted on the sample sizes included in the analysis. Collection of outcome data was also challenging particularly during the initial phase of the virtual programmes. Operationally, exit data was either collected in person or participants were emailed or mailed an exit questionnaire or, more recently, were sent a link to complete the questionnaire online. Non‐completion of exit questionnaires was problematic and follow‐up of participants to remind them to do so was prohibitively burdensome on the evaluation team. Baseline questionnaires were completed by a dietitian and participants at the initial assessment, so the quality of the initial data was excellent. However, little directive support was offered for the completion of the exit questionnaire which led to some questions being unanswered or sections of the questionnaire incomplete and so the quality of the outcome data impacted on the quantity and quality of the data used in the analysis.

## 5 Conclusion

The SELHLP demonstrated meaningful clinical and behavioural benefits for participants who completed the programme, with both Balance and Kickstart interventions achieving clinically significant weight loss across diverse delivery models. However, the TMR approach used in Kickstart produced considerably greater weight loss. Virtual delivery notably enhanced outcomes in the Balance programme, while weight loss among Kickstart participants was consistent across delivery modes. However, the overall completion rate was modest, 43%, and the primary outcome for ≥ 5% weight loss among 70% of completers was not met in either the virtual or face‐to‐face programmes. This mirrors trends from other comparable Tier 3 services. Importantly, the analysis highlighted persistent inequalities in outcomes by ethnicity and sex, with Black participants and women less likely to achieve significant weight loss, underscoring the need for more culturally relevant and tailored interventions. Adaptation and evaluation of weight management services to address health inequalities, improve programme engagement and optimise outcomes for all population groups remain essential.

## Author Contributions

**Majella O'Keeffe:** conceptualisation, data curation, formal analysis, methodology, original draft preparation. **Emiliano Pena‐Altamira:** project administration; data curation, original draft preparation, review and editing. **Sumaya Shuriye:** project administration; review and editing. **Danielle Dunk:** project administration; data curation, review and editing. **Oliver Canfell:** review and editing. **Rhys White:** review and editing. **Alastair Duncan:** conceptualisation, review and editing.

## Funding

This service evaluation was funded by Guys and St Thomas' NHS Foundation Trust. The service evaluation was independently led by the corresponding author. The funder had no role in the design of the evaluation, analysis, interpretation of data or the writing of the manuscript.

## Ethics Statement

This project was conducted as a quality improvement service evaluation using anonymised data and did not require NHS ethical approval. Approval for data use and analysis was obtained from the Quality Improvement and Patient Safety committee at GSTT (Reference 14211).

## Conflicts of Interest

The authors declare no conflicts of interest.

## Supporting information


**Table S1:** Impact of Balance and Kickstart on weight loss by ethnicity.

**Table S2:** Univariable binary regression models for predictors of ≥ 5% weight loss among programme completers for Balance and Kickstart.

**Table S3:** Impact of Balance and Kickstart on secondary outcomes for programme completers of the Southeast London Healthy Living programme. Data is presented as *N* (%) or median (IQR). For clinical familiarity, means (SD) are also reported.

## Data Availability

The data presented are not publicly available due to the fact that the data were collected as part of a clinical service. Reasonable requests for the data can be made via the corresponding author but the release of the data will be subject to approval by the clinical service leads and the Quality Improvement and Patient Safety oversight committee.
