# Supplementary material for: Impact of and Comparative Outcomes for Digital and In‐Person Interventions for Complex Obesity in a Diverse Urban Population
Source: Endocrinol Diabetes Metab. 2026 Apr 3;9(3):e70215. doi: 10.1002/edm2.70215 (PMC13051926; doi:10.1002/edm2.70215)
Supplement: Supplementary file 1 — Table S1: Impact of Balance and Kickstart on weight loss by ethnicity. [file EDM2-9-e70215-s003.docx]

| **Supplemental Table 1:** Impact of Balance and Kickstart on weight loss by ethnicity. | | | | | | | | | | |
| --- | --- | --- | --- | --- | --- | --- | --- | --- | --- | --- |
|  | **White** | | **Black African/ Caribbean** | | **Other** | | **p-value^1^** | **White V Black African/ Caribbean^2^** | **White vs Other^2^** | **Black African/ Caribbean vs Other^2^** |
|  | **N** | **Mean (SD)** | **N** | **Mean (SD)** | **N** | **Mean (SD)** |  |  |  |  |
| **Balance Combined** |  |  |  |  |  |  |  |  |  |  |
| Initial weight, kg | 273 | 126.1 (19.4) | 176 | 122.4 (19) | 96 | 122.4 (22.3) |  |  |  |  |
| 12-mo weight, kg | 226 | 120.5 (20.6) | 150 | 121.7 (21.6) | 84 | 117.6 (22.6) |  |  |  |  |
| Weight change, kg | 226 | -4.8 (10.2) | 150 | -1.7 (8.3) | 83 | -4.4 (11.3) | <0.001 | Z=-4.315, p<0.001 | Z=-1.051, p=0.293 | Z=-2.163, p=0.031 |
| Weight change, % | 226 | -3.7 (8) | 150 | -1.4 (6.3) | 83 | -3.5 (8.4) |  |  |  |  |
| **Balance F2F** |  |  |  |  |  |  |  |  |  |  |
| Initial weight, kg | 164 | 127 (20.2) | 106 | 121.1 (19) | 52 | 120.7 (21) |  |  |  |  |
| 12-mo weight, kg | 134 | 122.5 (21.8) | 92 | 122.3 (21) | 47 | 116.3 (21.8) |  |  |  |  |
| Weight change, kg | 134 | -4 (8.9) | 92 | -0.2 (6) | 46 | -4.7 (10.1) | <0.001 | Z=-4.323, p<0.001 | Z=-0.139, p=0.889 | Z=-3.272, p=0.001 |
| Weight change, % | 134 | -3.3 (7) | 92 | 0.2 (4.6) | 46 | -3.8 (7.2) |  |  |  |  |
| **Balance V** |  |  |  |  |  |  |  |  |  |  |
| Initial weight, kg | 102 | 124.9 (18.1) | 70 | 124.4 (19.2) | 44 | 124.6 (23.7) |  |  |  |  |
| 12-mo weight, kg | 92 | 117.5 (18.4) | 56 | 121 (23.3) | 37 | 119.4 (23.8) |  |  |  |  |
| Weight change, kg | 92 | -5.9 (11.9) | 58 | -4.1 (10.7) | 37 | -4 (12.8) | 0.2 | Z=-1.538, p=0.124 | Z=-1.372, p=0.17 | Z=0.218, p=0.828 |
| Weight change, % | 92 | -4.4 (10.1) | 58 | -3.4 (8.1) | 37 | -3 (9.7) |  |  |  |  |
| **Kickstart Combined** |  |  |  |  |  |  |  |  |  |  |
| Initial weight, kg | 170 | 121 (18.2) | 108 | 122.5 (18.6) | 42 | 120 (20) |  |  |  |  |
| 12-mo weight, kg | 142 | 107.3 (18.4) | 86 | 117.5 (21.7) | 38 | 105 (19.4) |  |  |  |  |
| Weight change, kg | 142 | -13.6 (13.6) | 86 | -5.6 (9) | 37 | -13.1 (14.7) |  |  |  |  |
| Weight change, % | 142 | -11 (10.5) | 86 | -4.7 (6.8) | 37 | -10.6 (10.7) | <0.001 | Z=-4.215, p<0.001 | Z=-2.744, p=0.006 | Z=-0.071, p=0.943 |
| **Kickstart F2F** |  |  |  |  |  |  |  |  |  |  |
| Initial weight, kg | 102 | 119 (18.3) | 50 | 121.3 (15.3) | 15 | 116.5 (23) |  |  |  |  |
| 12-mo weight, kg | 84 | 106.3 (18.5) | 40 | 116.5 (15.2) | 15 | 98 (18.6) |  |  |  |  |
| Weight change, kg | 84 | -13 (13.1) | 40 | -5.1 (7.4) | 14 | -15.6 (16.2) | <0.001 | Z=-4.125, p<0.001 | Z=-0.71, p=0.943 | Z=-2.744, p=0.006 |
| Weight change, % | 84 | -10.5 (10.6) | 40 | -4 (6) | 14 | -12.8 (11.1) |  |  |  |  |
| **Kickstart V** |  |  |  |  |  |  |  |  |  |  |
| Initial weight, kg | 15 | 116.5 (23) | 58 | 123.6 (21.2) | 27 | 121.8 (17.8) |  |  |  |  |
| 12-mo weight, kg | 15 | 98 (18.6) | 46 | 118.5 (26.2) | 23 | 109.2 (19) |  |  |  |  |
| Weight change, kg | 14 | -15.6 (16.2) | 46 | -6.1 (10.1) | 23 | -11.5 (13.9) | 0.018 | Z=-2.795, p=0.005 | Z=-0.382, p=0.702 | Z=-1.680, p=0.093 |
| Weight change, % | 14 | -12.8 (11.2) | 46 | -5.2 (7.5) | 23 | -9.3 (10.4) |  |  |  |  |
| ^1^ Kruskal Wallis; ^2^ Mann Whitney U. Statistical significance p<0.005 | | | | | | | | | | |
