# Supplementary material for: Impact of and Comparative Outcomes for Digital and In‐Person Interventions for Complex Obesity in a Diverse Urban Population
Source: Endocrinol Diabetes Metab. 2026 Apr 3;9(3):e70215. doi: 10.1002/edm2.70215 (PMC13051926; doi:10.1002/edm2.70215)
Supplement: Supplementary file 2 — Table S2: Univariable binary regression models for predictors of ≥ 5% weight loss among programme completers for Balance and Kickstart. [file EDM2-9-e70215-s002.docx]

**Supplemental Table 2:** Univariable binary regression models for predictors of ≥5% weight loss among programme completers for Balance and Kickstart.

|  | **Balance combined** | | | | | | | |
| --- | --- | --- | --- | --- | --- | --- | --- | --- |
|  | **N (%)** | **Β (SE)** | **Wald** | **df** | **OR (95% CI)** | **P-value** | **Cox & Snell R^2^** | **Nagelkerke R^2^** |
| **Sex** | 470 |  |  |  |  |  | 0.018 | 0.024 |
| Male (ref) |  | - | - | - | 1 | - |  |  |
| Female |  | -0.718 (0.246) | 8.525 | 1 | 0.488 (0.301-0.79) | 0.004 |  |  |
| **Ethnicity** | 459 |  |  |  |  |  | 0.037 | 0.05 |
| White (ref) |  | - | - | - | 1 | - |  |  |
| Black |  | -0.912 (0.224) | 16.522 | 1 | 0.402 (0.259-0.624) | <0.001 |  |  |
| Other |  | -0.365 (0.26) | 1.978 | 1 | 0.694 (0.417-1.155) | 0.16 |  |  |
| **Relationship status** | 294 |  |  |  |  |  | 0.013 | 0.017 |
| Single (ref) |  | - | - | - | 1 | - |  |  |
| Married/civil partnership |  | 0.466 (0.26) | 3.21 | 1 | 1.593 (0.957-2.652) | 0.073 |  |  |
| Separated^&^ |  | -0.084 (0.364) | 0.054 | 1 | 0.919 (0.45-1.877) | 0.817 |  |  |
| **Deprivation^^^** | 457 |  |  |  |  |  | 0.009 | 0.011 |
| High |  | - | - | - | 1 | - |  |  |
| Moderate |  | 0.098 (0.234) | 0.178 | 1 | 1.103 (0.698-1.744) | 0.673 |  |  |
| Low |  | 0.453 (0.231) | 3.838 | 1 | 1.573 (1-2.475) | 0.05 |  |  |
| **Age** | 471 | 0.016 (0.007) | 4.78 | 1 | 1.017 (1.002-1.032) | 0.029 | 0.01 | 0.014 |
| **Weight at baseline, kg** | 471 | 0.006 (0.005) | 1.27 | 1 | 1.006 (0.996-1.015) | 0.26 | 0.004 | 0.004 |
|  | **Kickstart combined** | | | | | | | |
|  | **N (%)** | **Β (SE)** | **Wald** | **df** | **OR (95% CI)** | **P-value** | **Cox & Snell R^2^** | **Nagelkerke R^2^** |
| **Sex** | 268 |  |  |  |  |  | 0.055 | 0.076 |
| Male (ref) |  | - | - | - | 1 | - |  |  |
| Female |  | -1.825 (0.454) | 16.185 | 1 | 0.161 (0.066-0.392) | <0.001 |  |  |
| **Ethnicity** | 265 |  |  |  |  |  | 0.082 | 0.112 |
| White (ref) |  | - | - | - | 1 | - |  |  |
| Black |  | -0.995 (0.284) | 12.228 | 1 | 0.37 (0.212-0.646) | <0.001 |  |  |
| Other |  | 0.557 (0.45) | 1.534 | 1 | 1.745 (0.723-4.212) | 0.216 |  |  |
| **Relationship status** | 239 |  |  |  |  |  | 0.015 | 0.021 |
| Single (ref) |  | - | - | - | 1 | - |  |  |
| Married/civil partnership |  | 0.522 (0.294) | 3.149 | 1 | 1.686 (0.947-3.003) | 0.076 |  |  |
| Separated^&^ |  | 0.018 (0.428) | 0.002 | 1 | 1.018 (0.44-2.354) | 0.967 |  |  |
| **Deprivation^^^** | 258 |  |  |  |  |  | 0.014 | 0.019 |
| High |  | - | - | - | 1 | - |  |  |
| Moderate |  | 0.022 (0.334) | 0.004 | 1 | 1.023 (0.531-1.968) | 0.947 |  |  |
| Low |  | 0.52 (0.303) | 2.949 | 1 | 1.681 (0.929-3.042) | 0.086 |  |  |
| **Age** | 268 | 0.036 (0.011) | 10.982 | 1 | 1.037 (1.015-1.06) | <0.001 | 0.043 | 0.058 |
| **Weight at baseline, kg** | 268 | 0.11 (0.007) | 2.266 | 1 | 1.011 (0.997, 1.025) | 0.132 | 0.009 | 0.012 |
| ^Based on index of multiple deprivation decile. ^&^Separated/Divorced/Widowed | | | | | | | | |
