# Supplementary material for: Impact of and Comparative Outcomes for Digital and In‐Person Interventions for Complex Obesity in a Diverse Urban Population
Source: Endocrinol Diabetes Metab. 2026 Apr 3;9(3):e70215. doi: 10.1002/edm2.70215 (PMC13051926; doi:10.1002/edm2.70215)
Supplement: Supplementary file 3 — Table S3: Impact of Balance and Kickstart on secondary outcomes for programme completers of the Southeast London Healthy Living programme. Data is presented as N (%) or median (IQR). For clinical familiarity, means (SD) are also reported. [file EDM2-9-e70215-s001.docx]

| **Supplemental table 3**: Impact of Balance and Kickstart on secondary outcomes for programme completers of the Southeast London Healthy Living programme. Data is presented as N(%) or median (IQR). For clinical familiarity, means (SD) are also reported. | | | | | | | | |
| --- | --- | --- | --- | --- | --- | --- | --- | --- |
|  | **Balance combined** | | | | **Kickstart combined** | | | |
|  | **N** | **Baseline**  **Median (IQR)** | **End of Programme**  **Median (IQR)** | **Z-value, P-value** | **N** | **Baseline**  **Median (IQR)** | **End of Programme**  **Median (IQR)** | **Z-value, P-value** |
| **Lipids** |  |  |  |  |  |  |  |  |
| Total cholesterol | 153 | 4.6 (1.5) | 4.5 (1.4) | -1.542, p=0.123 | 66 | 4.8 (1.4) | 4.5 (1.1) | -1.176, p=0.24 |
| LDL-c | 109 | 2.5 (1.2) | 2.4 (1.6) | -1.767, p=0.077 | 49 | 2.5 (1.3) | 2.2 (1.5) | -1.746, p=0.081 |
| HDL-c | 120 | 1.3 (0.5) | 1.3 (0.5 | -1.497, p=0.134 | 55 | 1.3 (0.4) | 1.4 (0.6) | -2.05, p=0.04 |
| **Blood Pressure** |  |  |  |  |  |  |  |  |
| SBP | 187 | 136 (17) | 132 (14) | -1.756, p=0.079 | 67 | 132 (23) | 132 (16) | -0.086, p=0.932 |
| DBP | 186 | 82 *11) | 80 (12) | -1.83, p=0.067 | 67 | 82 (11) | 82 (14) | -0.003, p=0.997 |
| **Glycaemic control** |  |  |  |  |  |  |  |  |
| HbA1c | 193 | 45 (16) | 43 (15) | -0.062, p=0.951 | 80 | 45 (12) | 43 (15) | -2.218, p=0.027 |
| **Dietary Quality & Eating Behaviours** |  |  |  |  |  |  |  |  |
| **Dietary quality** | 297 | 1.7 (0.7) | 1.6 (0.6) | -3.081, p=0.002 | 133 | 1.5 (0.6) | 1.4 (0.6) | -2.179, p=0.029 |
| **BED*** | 117 | 1 (2) | 1 (2) | -2.781, p=0.005 | 74 | 1 (1) | 1 (3.25) | -3.254, p=0.001 |
| **Eating behaviours^^^** | 92 | 8 (6) | 8 (6.8) | -0.61, p=0.543 | 78 | 7 (5.3) | 7 (4) | -0.66, p=0.509 |
|  |  | **Mean (SD)** | **Mean (SD)** |  |  | **Mean (SD)** | **Mean (SD)** |  |
| **Lipids** |  |  |  |  |  |  |  |  |
| Total cholesterol | 153 | 4.7 (1.1) | 4.5 (1) |  | 66 | 4.7 (1.1) | 4.6 (1) |  |
| LDL-c | 109 | 2.6 (1) | 2.5 (1) |  | 49 | 2.5 (1) | 2.3 (1) |  |
| HDL-c | 120 | 1.4 (0.5) | 1.4 (0.4) |  | 55 | 1.5 (0.6) | 1.6 (0.6) |  |
| **Blood Pressure** |  |  |  |  |  |  |  |  |
| SBP | 187 | 135 (15) | 132 (14) |  | 67 | 132 (15) | 133 (15) |  |
| DBP | 186 | 82 (10) | 81 (10) |  | 67 | 82 (9) | 82 (14) |  |
| **Glycaemic control** |  |  |  |  |  |  |  |  |
| HbA1c | 193 | 50 (15) | 49 (17) |  | 80 | 49 (19) | 50 (21) |  |
| **Dietary Quality & Eating Behaviours** | | |  |  |  |  |  |  |
| **Dietary quality** | 297 | 1.9 (0.5) | 1.6 (0.4) |  | 133 | 1.5 (0.4) | 1.4 (0.4) |  |
| **BED*** | 117 | 1.5 (1.6) | 2 (1.8) |  | 74 | 1.1 (1.3) | 1.7 (1.7) |  |
| **Eating behaviours^^^** | 92 | 8 (3.1) | 7.6 (3.5) |  | 78 | 7.1 (3.2) | 7.3 (3) |  |
|  |  | **N (%)** | **N (%)** |  |  | **N (%)** | **N (%)** |  |
| **Physical Activity Index** | | **128** | **128** |  |  | **64** | **64** |  |
| Inactive |  | 41 (32) | 40 (31) | X2=53.753, df(9), p<0.001 |  | 20 (31) | 14 (22) | X2=20.84, df(9),  p=0.053 |
| Mod inactive |  | 38 (30) | 23 (18) |  |  | 19 (30) | 20 (31) |  |
| Mod active |  | 21 (16) | 28 (22) |  |  | 14 (22) | 20 (13) |  |
| Active |  | 28 (22) | 37 (29) |  |  | 11 (17) | 16 (25) |  |
| **Physical exercise** |  | **294** | **294** |  |  | **149** | **149** |  |
| None |  | 225 (77) | 171 (58) | X2=62.788, df(9), p<0.001 |  | 108 (73) | 74 (48) | X2=16.82, df(9),  p=0.052 |
| <1 hour |  | 23 (8) | 42 (14) |  |  | 12 (8) | 26 (17) |  |
| 1-3 hours |  | 27 (9) | 59 (20) |  |  | 18 (12) | 34 (23) |  |
| >3 hours |  | 19 (7) | 22 (8) |  |  | 11 (7) | 18 (12) |  |
| **Walking** |  | **296** | **296** |  |  | **148** | **148** |  |
| None |  | 23 (8) | 22 (7) | X2=26.225, df(9), p=0.002 |  | 10 (7) | 13 (9) | X2=11.04, df(9),  p=0.273 |
| <1 hour |  | 78 (26) | 80 (27) |  |  | 27 (18) | 37 (25) |  |
| 1-3 hours |  | 94 (32) | 106 (36) |  |  | 42 (28) | 44 (30) |  |
| >3 hours |  | 101 (34) | 88 (30) |  |  | 69 (47) | 54 (37) |  |
| *BED, binge eating disorder which was assessed using the Questionnaire on Eating and Weight Patterns-Revised (QEWPR). ^^^Eating behaviours were assessed using the Three Factor Easting Questionnaire (TFEQ). **Mod active/inactive, moderately active/inactive. | | | | | | | | |
